# Antioxidant enzyme responses in different wheat species infested with the corn leaf aphid, *Rhopalosiphum maidis* Fitch

**DOI:** 10.3389/fpls.2025.1693782

**Published:** 2025-10-31

**Authors:** Pritam Kumari, Poonam Jasrotia, Sunny Maanju, Sindhu Sareen, Dinesh Kumar

**Affiliations:** ^1^ Crop Protection Section, Indian Council of Agricultural Research (ICAR)- Indian Institute of Wheat and Barley Research, Karnal, Haryana, India; ^2^ Department of Entomology, Chaudhary Charan Singh (CCS) Haryana Agricultural University, Hisar, Haryana, India

**Keywords:** cereals, host plant resistance, biochemical markers, aphid resistance breeding, antioxidant enzymes

## Abstract

Wheat (*Triticum aestivum* L.) is a staple crop worldwide, but it remains vulnerable to the corn leaf aphid (*Rhopalosiphum maidis* Fitch), a major pest that causes both direct yield losses and indirect damage through disease transmission. To elucidate biochemical mechanisms underlying resistance, 65 wild and synthetic wheat genotypes were evaluated under aphid-infested and uninfested conditions. Aphid nymphal mortality varied significantly across genotypes, with amphidiploid and *Aegilops kotschyi* showing the highest resistance, while synthetic wheat lines exhibited moderate aphid mortality. Biochemical assays revealed consistent induction of antioxidant enzymes, viz., catalase (CAT), ascorbate peroxidase (APX), peroxidase (POX), and glutathione reductase (GR), across all genotypes upon infestation. Synthetic wheat displayed the highest enzymatic activities, indicating robust oxidative stress tolerance, whereas amphidiploid wheat maintained lower enzyme activity but exerted strong aphid mortality, suggesting reliance on non-enzymatic or constitutive defenses. Additionally, phenylalanine ammonia-lyase (PAL) and polyphenol oxidase (PPO), key enzymes in the phenylpropanoid pathway, were strongly upregulated in synthetic wheat and *Ae. kotschyi*, highlighting their role in secondary metabolite-mediated defense. These findings demonstrate that wheat resistance to *R. maidis* is multifaceted, involving both antioxidant enzyme regulation and phenylpropanoid metabolism. Genotypic differences underscore the potential of wild relatives and synthetic wheats as valuable genetic resources for breeding durable, eco-friendly aphid-resistant wheat cultivars. Integrating these biochemical insights into breeding programs can accelerate the development of resistant cultivars, reducing pesticide use and strengthening food security under pest and climate challenges.

## Introduction

Wheat (*Triticum aestivum* L.) is one of the most important cereal crops, serving as a staple food for nearly 40% of the global population (Ahmad et al., 2024; Wei et al., 2022). Its global importance lies not only in meeting caloric requirements and eradicating hunger but also in providing essential proteins, carbohydrates, and micronutrients. However, wheat productivity is under constant threat from various biotic and abiotic stresses that severely affect yield stability (Ahmad et al., 2022). Among the biotic constraints, insect pests represent a major challenge to wheat cultivation across different agroecological regions. Aphids, in particular, have emerged as one of the most destructive insect groups in wheat due to their ability to rapidly colonize crops, directly extract nutrients, and serve as efficient vectors of plant pathogens (Jasrotia et al., 2022b).

The corn leaf aphid, *Rhopalosiphum maidis* Fitch (Hemiptera: Aphididae), is considered one of the most damaging cereal aphid species, with widespread distribution and high adaptability across tropical and subtropical regions (Csorba et al., 2024). This pest is capable of causing direct yield losses of 20%–30% through phloem sap feeding, which weakens plants, reduces photosynthetic efficiency, and limits grain filling (Singh and Jasrotia, 2020). In addition, it causes indirect losses ranging from 5% to 80% by transmitting viral and fungal diseases (Aradottir and Crespo-Herrera, 2021). Thus, *R. maidis* is considered a major threat to wheat production globally, particularly in regions where climatic conditions favor its proliferation (Yadav et al., 2024).

Current management strategies for aphids largely rely on the use of systemic insecticides, including neonicotinoids and organophosphates (Devrani et al., 2018; Katare et al., 2021; Kaleem Ullah et al., 2023). While being very effective in the short term, continuous, indiscriminate, and long-term application of pesticides has led to a series of negative consequences such as development of insecticide resistance, resurgence of aphid populations, secondary pest outbreaks, disruption of natural enemy populations, contamination of soil and water ecosystems, and potential health risks to humans and livestock (Liu et al., 2018; Saroop and Tamchos, 2024). These drawbacks underscore the urgent need for environmentally sustainable and ecologically sound approaches for aphid management (Araújo et al., 2023). Among the alternatives, host plant resistance is considered the most promising, economical, and eco-friendly strategy (Jasrotia et al., 2021; Radchenko et al., 2022; Singh et al., 2022). Resistance traits are often linked to morphological, biochemical, and physiological characteristics of the plant, which together determine the level of susceptibility or tolerance to the pest attack (Niraz et al., 1985; Ciepiela, 1989; Smith and Chuang, 2014; Manikanda Boopathi and Shobhana, 2024).

Plant–insect interactions are the outcome of long-term co-evolutionary processes, resulting in complex defense and counterdefense mechanisms. Plants deploy both constitutive and inducible defenses to protect themselves against herbivores (Leitner et al., 2005; Prabhakaran et al., 2025). Constitutive defenses, such as trichomes, cuticular waxes, and pre-formed secondary metabolites, are continuously present regardless of the stress (Al-Khayri et al., 2023). In contrast, induced defenses are activated upon insect attack and often involve biochemical changes mediated by signaling pathways (Mostafa et al., 2022). These induced responses are highly dynamic and can lead to the synthesis of toxic or deterrent compounds, structural reinforcement, or alterations in primary and secondary metabolism (Zaynab et al., 2018; Zhang et al., 2022).

One of the earliest responses of plants to insect feeding is the generation of reactive oxygen species (ROS), including hydrogen peroxide (H_2_O_2_), superoxide radicals (O_2_^−^), and hydroxyl radicals (OH) (Dhiman et al., 2025). Although ROS function as important signaling molecules, their excessive accumulation results in oxidative stress, leading to damage of lipids, proteins, and nucleic acids (Xie et al., 2016; El-Zohri et al., 2020). To mitigate such damage, plants activate an antioxidant defense system comprising enzymatic and non-enzymatic components. Non-enzymatic elements include phenolic compounds, flavonoids, tannins, and alkaloids, which deter herbivory and interfere with insect physiology (Debona et al., 2012; Smith and Clement, 2012; Raza et al., 2021; Dash et al., 2025). The enzymatic machinery includes key oxidative enzymes such as peroxidase (POX/POD), polyphenol oxidase (PPO), phenylalanine ammonia-lyase (PAL), and catalase, which scavenge ROS and facilitate the biosynthesis of defensive secondary metabolites (Taggar et al., 2012; Sha et al., 2015).

Peroxidases (POX/POD) play dual roles in plant defense, as they participate in lignin formation and suberization, strengthening cell walls against insect penetration, and they detoxify ROS, thereby enhancing oxidative stress tolerance (Usha Rani and Jyothsna, 2010). Polyphenol oxidases (PPOs) catalyze the oxidation of phenolic compounds to quinones, which can polymerize to form protective barriers or interfere with insect digestion. Increased PPO activity has been observed in several crops under aphid infestation, including sorghum and wheat (Chang et al., 2008). PAL is a key enzyme of the phenylpropanoid pathway, converting L-phenylalanine into trans-cinnamic acid, the precursor of diverse phenolic compounds and phytoalexins involved in defense (Dixon et al., 2002; Chaman et al., 2003). Elevated PAL activity has been documented in cotton leaves under *Aphis gossypii* attack and in wheat infested by *Sitobion avenae*, with resistant cultivars consistently showing higher PAL activity compared to the susceptible ones (Li et al., 2008; Han et al., 2009; Xu et al., 2021). Thus, these enzymes are widely recognized as biochemical markers of insect resistance. Similarly, aphid feeding triggered significant induction of antioxidant and defense-related enzymes in wheat, highlighting their key role in herbivory tolerance. Genotypic differences in enzyme activity indicated variable defensive capacity, with some varieties exhibiting reduced susceptibility under specific conditions (Kaur et al., 2017).

In addition to enzymatic changes, aphid infestation influences plant secondary metabolite accumulation, nutrient allocation, and structural properties. Resistant wheat genotypes often exhibit higher phenolic content, altered carbohydrate composition, and stronger structural barriers such as increased leaf thickness and trichome density (Kessler et al., 2004; Demis, 2024). These traits act synergistically with biochemical defenses to reduce aphid feeding efficiency, digestion, and reproduction. However, aphids, in turn, have evolved adaptive mechanisms, including salivary effectors that suppress plant defense signaling and manipulation of host metabolism to enhance nutrient availability (Zaynab et al., 2018). This evolutionary arms race makes it imperative to understand not only the static defense traits but also the dynamic biochemical responses that occur during pest infestation.

Despite significant progress in understanding plant–aphid interactions, there remain critical gaps in knowledge regarding the biochemical basis of wheat resistance to *R. maidis*. While several studies have reported enhanced enzyme activities under aphid infestation in different crops, limited information is available on the temporal changes in antioxidant enzymes and secondary metabolites in wheat (Xu et al., 2021; Zhang et al., 2022; Guan et al., 2022). Furthermore, little is known about how these responses differ between resistant and susceptible wheat genotypes, especially those derived from wild and synthetic backgrounds. Wild relatives of wheat are known to harbor diverse resistance genes and biochemical traits, making them valuable resources for broadening the genetic base of resistance in cultivated wheat.

Therefore, the present investigation was designed with the following objectives: i) to evaluate the biochemical responses of wheat genotypes under aphid stress, with emphasis on antioxidant enzymes such as POD, PPO, and PAL; ii) to compare the differential responses between resistant and susceptible cultivars, including wild and synthetic wheat lines; and iii) to identify key biochemical markers that may serve as indicators of resistance to *R. maidis*. Understanding these biochemical interactions will not only advance our knowledge of plant defense mechanisms but also provide valuable insights for breeding durable aphid-resistant wheat varieties through conventional and molecular approaches.

## Material and methods

### Plant material and experimental setup

A total of 65 wild and synthetic wheat genotypes (**Table 1**) were obtained from the Germplasm Resource Unit (GRU) facility of ICAR-Indian Institute of Wheat and Barley Research (IIWBR), Karnal (India), and were used for determining the biochemical profiles. These genotypes included seven species of wheat, namely, *Aegilops tauschii*, *Ae. kotschyi*, *Ae. ovata*, *Ae. peregrina*, synthetic wheat, amphidiploid, and *Triticum dicoccoides.*

**Table 1:** List of wild and synthetic wheat genotypes used for biochemical analysis.

Sr. no.	Species	Accession no.	Sr. no.	Species	Accession no.	Sr. no.	Species	Accession no.	Sr. no.	Species	Accession no.
1	*Aegilops tauschii*	AetaNA1	18	*Ae. tauschii*	14336a	35	*Ae. tauschii*	16	52	*Ae. peregrina*	PI 604168
2		13763a	19		59b	36		15a	53		PI 603931
3		3761a	20		14325b	37		13763b	54		55c
4		PI 554324	21		14336b	38		12b	55	*T. dicoccoides*	102b
5		9787a	22		3784a	39	*Ae. kotschyi*	3774a	56		13993a
6		12a	23		3757a	40		3774b	57		103a
7		14578a	24		62b	41	*Ae. ovata*	24a	58	Amphidiploid	ID 81/17
8		3761b	25		9822a	42		21b	59		EC 787010
9		1	26		3757b	43		23	60		EC 787008
10		13764b	27		62c	44	*Ae. peregrina*	PI 604192a	61	Synthetic	SYN9
11		14578b	28		3806a	45		PI 603247	62		SYN 5
12		9829b	29		9830	46		PI 604149	63		SYN74
13		62a	30		8b	47		PI 604150	64		SYN20
14		9803a	31		13765	48		PI 604176b	65		SYN87
15		14325a	32		3806b	49		55a			
16		2a	33		3	50		PI 604145b			
17		3744b	34		18	51		55b			

Biochemical studies were carried out under two conditions: unchallenged (without aphid infestation) and challenged (with aphid infestation). Wheat genotypes, identified by names and accession numbers, were sown in 1-m rows with 30 cm spacing, using a randomized block design (RBD) with three replications (two rows per replication). The experiment was conducted in a screenhouse maintained at 15°C–20°C, 60%–70% relative humidity, and natural light.

In the unchallenged condition (control), plants were protected with net covers to prevent insect attack. In the challenged condition (infested), plants were also net-covered (50-mesh size) but deliberately infested with aphids. To assess aphid mortality on each wheat genotype, 200 *R. maidis* nymphs from established corn leaf aphid cultures were released onto 15-day-old seedlings using a soft hairbrush. The seedlings were then enclosed in net cages to maintain uniform selection pressure. After 24 h, the number of nymphs settled on the plants was recorded, and all unsettled individuals were removed. Mortality was calculated by counting the number of dead aphids among the settled population and expressing it as a percentage for each accession. The mean mortality percentage across accessions was then used to determine species-level mortality rates, while surviving nymph counts were used to further validate mortality estimates. After that, the flag leaf samples were collected from infested as well as uninfested plants with the help of scissors during morning hours. Samples were collected in an ice-containing thermos and stored at−20°C in the refrigerator. The standard protocols were used for the estimation of the biochemical parameters.

To estimate the activities of antioxidative enzymes, viz., catalase, peroxidase, ascorbate peroxidase, and glutathione reductase, 0.5 g of leaf tissue was ground in a mortar and pestle with 5.0 mL of 100 mM potassium phosphate buffer (pH 7.0) under ice-cold conditions. The homogenate was then centrifuged at 15,000×*g* for 20 min, and the resulting supernatant (crude protein extract) was used to assay the enzyme activities.

### Catalase activity

Catalase activity was measured by following the method of Aebi (1984). The method of choice for biological material was the UV-spectrophotometric method. The rate of decomposition of H_2_O_2_ was followed by a decrease in absorbance at 240 nm in a reaction mixture containing 1.5 mL of 100 mM phosphate buffer (pH 7.0), 1.2 mL of 150 mM hydrogen peroxide, and 0.3 mL of enzyme extract. One unit of enzyme activity is calculated as the amount of enzyme required to liberate half the peroxide oxygen from H_2_O_2_. The decomposition of H_2_O_2_ was followed directly by the decrease in extinction per unit time at 240 nm. The difference in extinction per unit of time was a measure of catalase activity. The formula employed to calculate catalase (CAT) activity is mentioned below, where the Extinction coefficient = 6.93 × 10^−3^ mM^−1^ cm^−1^.


$$\begin{array}{c} Unit\;Activity(Units/min/g\;FW)\\ =\frac{\text{Change in absorbance}/\text{minute}\times \text{Total volume }(\text{ml})}{\text{Extinction coefficient}\times \text{Volume of sample taken }(\text{ml})} \end{array}$$

$$\begin{array}{c} CAT\;Specific\;Activity\;(mol\;UA/mg\;Protein)\\ =\frac{Unit\;Activity\;(Units/min/g\;FW)}{\text{Protein Content }(\text{mg}/\text{g FW})} \end{array}$$

### Ascorbate peroxidase activity

Ascorbate peroxidase activity was measured by following the methodology suggested by Nakano and Asada (1981) with slight modifications. It catalyzes the reduction of H_2_O_2_ using the substrate ascorbate. Three milliliters of the reaction mixture consisting of 1.5 mL of 100 mM phosphate buffer (pH 7.0), 300 µL of 5.0 mM ascorbate, 600 µL of 0.5 mM H_2_O_2_, and 600 µL of enzyme extract was taken, and the decrease in absorbance was recorded at 290 nm. One unit of the enzyme activity was calculated as the amount of enzyme required to oxidize 1.0 µM of ascorbate/min/g FW. The enzyme activity was calculated by using the equation given below, where the Extinction coefficient = 2.8 mM^−1^ cm^−1^.


$$\begin{array}{c} Unit\;Activity(Units/min/g\;FW)\\ =\frac{\text{Change in absorbance}/\text{minute}\times \text{Total volume }(\text{ml})}{\text{Extinction coefficient}\times \text{Volume of sample taken }(\text{ml})} \end{array}$$

$$\begin{array}{c} APX\;Specific\;Activity\;(mol\;UA/mg\;Protein)\\ =\frac{Unit\;Activity\;(Units/min/g\;FW)}{\text{Protein Content }(\text{mg}/\text{g FW})} \end{array}$$

### Peroxidase activity

The activity of POX can be determined by the decrease of H_2_O_2_ or the hydrogen donor or the formation of an oxidized compound. In this case, guaiacol is used as a substrate for the estimation of POX activity. POX activity was assayed following the slightly modified method of Putter (1974). In the test cuvette, the reaction mixture comprising 3.0 mL of 0.1 M phosphate buffer (pH 7.0), 50 µL of 20.0 mM guaiacol solution, 100 µL of enzyme sample, and 30 µL of 12.3 mM H_2_O_2_ solution was taken. The rate of formation of guaiacol dehydrogenation product (GDHP) was followed spectrophotometrically at 436 nm. One unit of enzyme activity was defined as the amount of enzyme catalyzing the formation of 1.0 µM of GDHP/min/g FW. The enzyme activity was calculated by using the equations given below, where the Extinction coefficient = 25 mM^−1^ cm^−1^.


$$\begin{array}{c} Unit\;Activity(Units/min/g\;FW)\\ =\frac{\text{Change in absorbance}/\text{minute}\times \text{Total volume }(\text{ml})}{\text{Extinction coefficient}\times \text{Volume of sample taken }(\text{ml})} \end{array}$$

$$\begin{array}{c} POX\;Specific\;Activity\;(mol\;UA/mg\;Protein)\\ =\frac{Unit\;Activity\;(Units/min/g\;FW)}{\text{Protein Content }(\text{mg}/\text{g FW})} \end{array}$$

### Glutathione reductase activity

For the estimation of activities of glutathione reductase, the method of Carlberg and Mannervik (1975) with slight modifications was used. In this method, 0.5 g of leaves were homogenized in a mortar and pestle with 5.0 mL of 100 mM potassium phosphate buffer at pH 7.0 under ice-cold conditions. The homogenate was centrifuged at 15,000×*g* for 20 min, and the supernatant was used for analysis of the activities of antioxidative enzymes. Glutathione reductase (GR) activity was determined by measuring the oxidation of NADPH at 340 nm in a reaction mixture containing 1.8 mL of 50 mM phosphate buffer (pH 7.6), 300 µL each of 3.0 mM EDTA, 1.0 mM of NADPH, 1.0 mM of oxidized glutathione (GSSG), and enzyme extract. The decrease in absorbance per minute was recorded at 340 nm. The GR activity was calculated by using the equations given below, where the Extinction coefficient = 6.22 mM^−1^ cm^−1^.


$$\begin{array}{c} Unit\;Activity(Units/min/g\;FW)\\ =\frac{\text{Change in absorbance}/\text{minute}\times \text{Total volume }(\text{ml})}{\text{Extinction coefficient}\times \text{Volume of sample taken }(\text{ml})} \end{array}$$

$$\begin{array}{c} GR\;Specific\;Activity\;(mmol\;UA/mg\;Protein)\\ =\frac{Unit\;Activity\;(Units/min/g\;FW)}{\text{Protein Content }(\text{mg}/\text{g FW})} \end{array}$$

### Phenylalanine ammonia-lyase activity

PAL activity was determined using a standard curve, prepared in the same manner as the enzyme assay, except that the extraction buffer was not included in the reaction mixture. Leaf tissue samples (1 g) were homogenized with 4 mL of chilled buffer Tris–HCl (50 mM) of pH 8.8, supplemented with 5% polyvinylpolypyrrolidone (PVP) and 0.01 mM of EDTA. Crude enzyme extract was obtained by subjecting the homogenate to centrifugation at 12,000×*g* for 20 min at 3°C. PAL activity was assayed following the slightly modified method of Ngadze et al. (2012). One milliliter of the supernatant was mixed with 2 mL of 0.05 M borate buffer (pH 8.8) and 1 mL of 0.02 M L-phenylalanine. The samples were incubated at 30°C for 1 h. The reaction was stopped by adding 0.5 mL of 10% trichloroacetic acid (TCA), and absorbance was read at 290 nm. Standard (t-cinnamic acid) was run in the range of 5–35 µg/mL. The enzyme activity is expressed as μg t-cinnamic acid formed/h/g FW tissue.

### Polyphenol oxidase activity

PPO activity was estimated as per the method of Archana et al. (2011) with slight modifications. A calibration curve was prepared following the same enzyme extraction protocol, using a reaction mixture consisting of all reagents in the same quantity except the extraction buffer. The activity of PPO in the test samples was then calculated using the calibration curve. A total of 100 mg of leaf sample was homogenized in a chilled mortar with 2 mL of 0.1 M sodium phosphate buffer (pH 7.0). The homogenate was centrifuged at 10,000×*g* at 4°C for 15 min, and the clear supernatant was used for the enzyme assay. A total of 0.2 mL of enzyme extract and 1.5 mL of 0.1 M sodium phosphate buffer (pH 6.5) was added, followed by 0.2 mL of 0.01 M catechol. The absorbance was recorded at 495 nm after an interval of 30 s up to 3 min.

### Statistical analysis

The statistical analysis was carried out in the RStudio version 4.4.3 (R Core Team, 2025). The nymphal mortality and enzyme activity data for CAT, ascorbate peroxidase (APX), POX, and GR were not normally distributed and were log-transformed before passing the Shapiro–Wilk test of normality. The Levene's test was carried out to test the homogeneity of variances. The nymphal mortality data were subjected to Welch's ANOVA, as the Levene's test was not satisfied (unequal variances), followed by the Games–Howell *post hoc* test for multiple comparison among the wheat species. The enzyme activity data of the control and infested group were separately subjected to one-way ANOVA followed by Tukey's HSD test based on estimated marginal means, but to compare the control and infested data for each wheat species individually for each enzyme, a two-way ANOVA was used, followed by pairwise *post hoc* comparisons using estimated marginal means and Sidak's adjustment to generate the significant letters. Data were also subjected to principal component analysis (PCA). All figures were also generated using RStudio version 4.4.3 (R Core Team, 2025).

## Results

**Figure 1:**
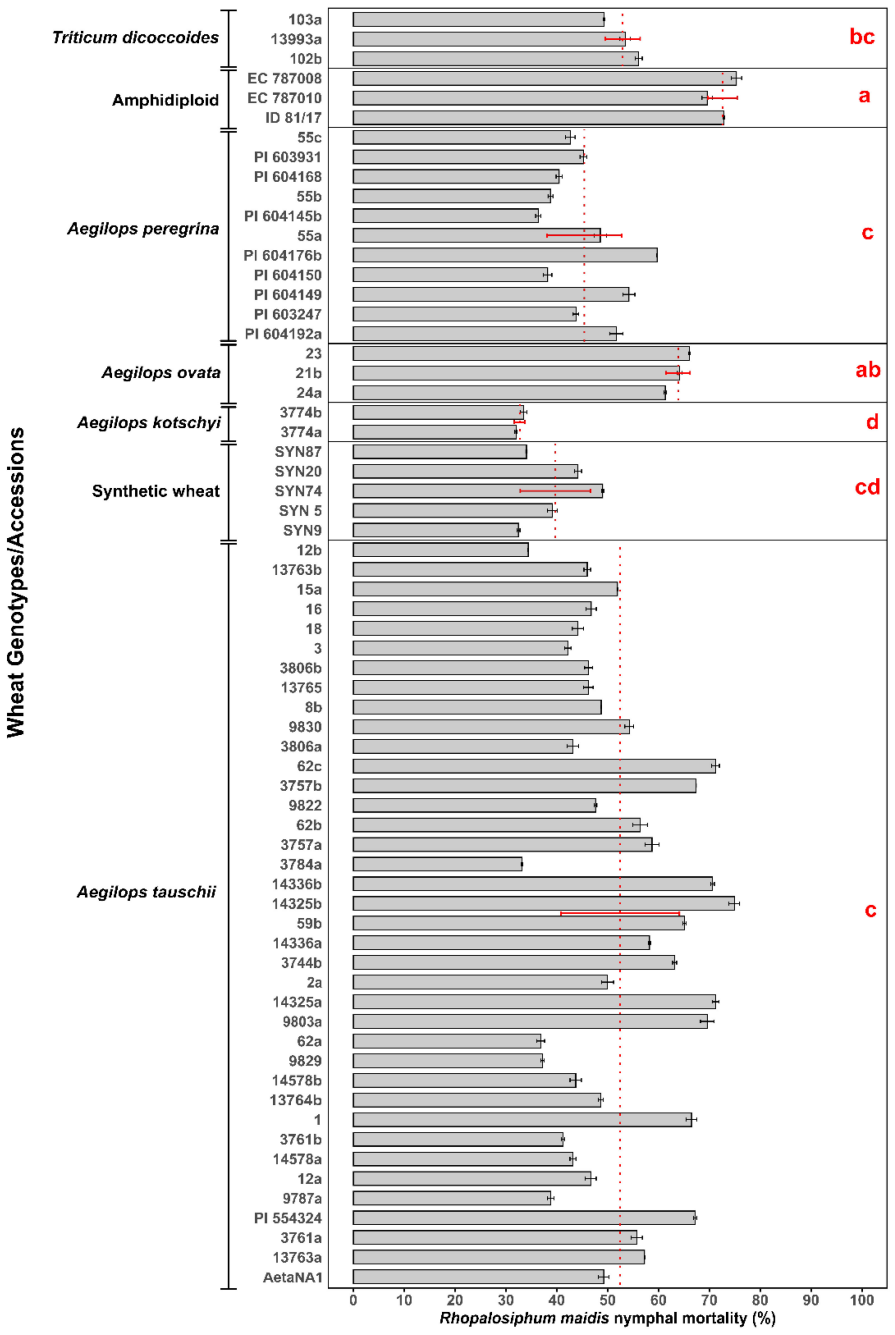
Mean percentage of *Rhopalosiphum maidis* nymphal mortality in different wild and synthetic wheat species (marked by a red dotted line with red standard deviation error bars) and their accessions (represented by gray bars with black standard deviation error bars). According to Games–Howell *post hoc* test, the lowercase letters in red represent significant differences among the means of different wheat species, with species sharing the same letter not having a significant difference.

The log-transformed mean percentage of *R. maidis* nymphal mortality varied significantly across different wild and synthetic wheat species (**Supplementary Table S1**) (*F*_6, 8.4_ = 88.456, *p* < 0.05) and their respective accessions/genotypes (**Figure 1**). Mortality rates ranged from 31.99% ± 0.22% in 3774a accession of *Ae. kotschyi* to 75.32% ± 1.02% in the most resistant accession EC 787008 of the amphidiploid. Among the species tested, amphidiploid wheat exhibited the highest nymphal mortality (72.56% ± 2.88%), which was significantly higher than all the other species groups except *Ae. ovata*, which showed 63.84% ± 2.37% nymphal mortality. *Triticum dicoccoides* also demonstrated relatively high mortality, with a mean percentage mortality of 52.94% ± 3.44%, followed by *Ae. tauschii* (52.46% ± 11.66%) and *Ae. peregrina* (45.41% ± 7.37%), which showed moderate levels of resistance. Synthetic wheat genotypes overall showed relatively lower mortality (39.75% ± 6.92%), whereas the lowest *R. maidis* nymphal mortality of 32.73% ± 1.04% was observed in *Ae*. *kotschyi*. Considerable variation was observed among the individual accessions of each species group. For instance, in *Ae. tauschii*, accession 14325b showed the highest mortality levels 74.91% ± 1.05%, while the lowest mortality of 33.12% ± 0.14% was observed in accession 3784a. Similarly, accessions of *Ae. peregrina* showed moderate but variable effects, with PI 604145b (36.34% ± 0.51%) demonstrating the lowest aphid mortality compared to the highest PI 604176b (59.72% ± 0.06%) among the *Ae. peregrina* accessions. The overall mean comparison confirmed significant differences among the wheat species. It is observed that resistance to *R. maidis* is species-dependent and varies even among accessions. These results highlight the potential of wild relatives, particularly amphidiploid, *Ae. ovata*, *Ae. peregrin*a, and *Ae. tauschii* accessions, as valuable genetic resources for breeding resistance against *R. maidis*.

### Catalase activity response to *Rhopalosiphum maidis* infestation

**Figure 2:**
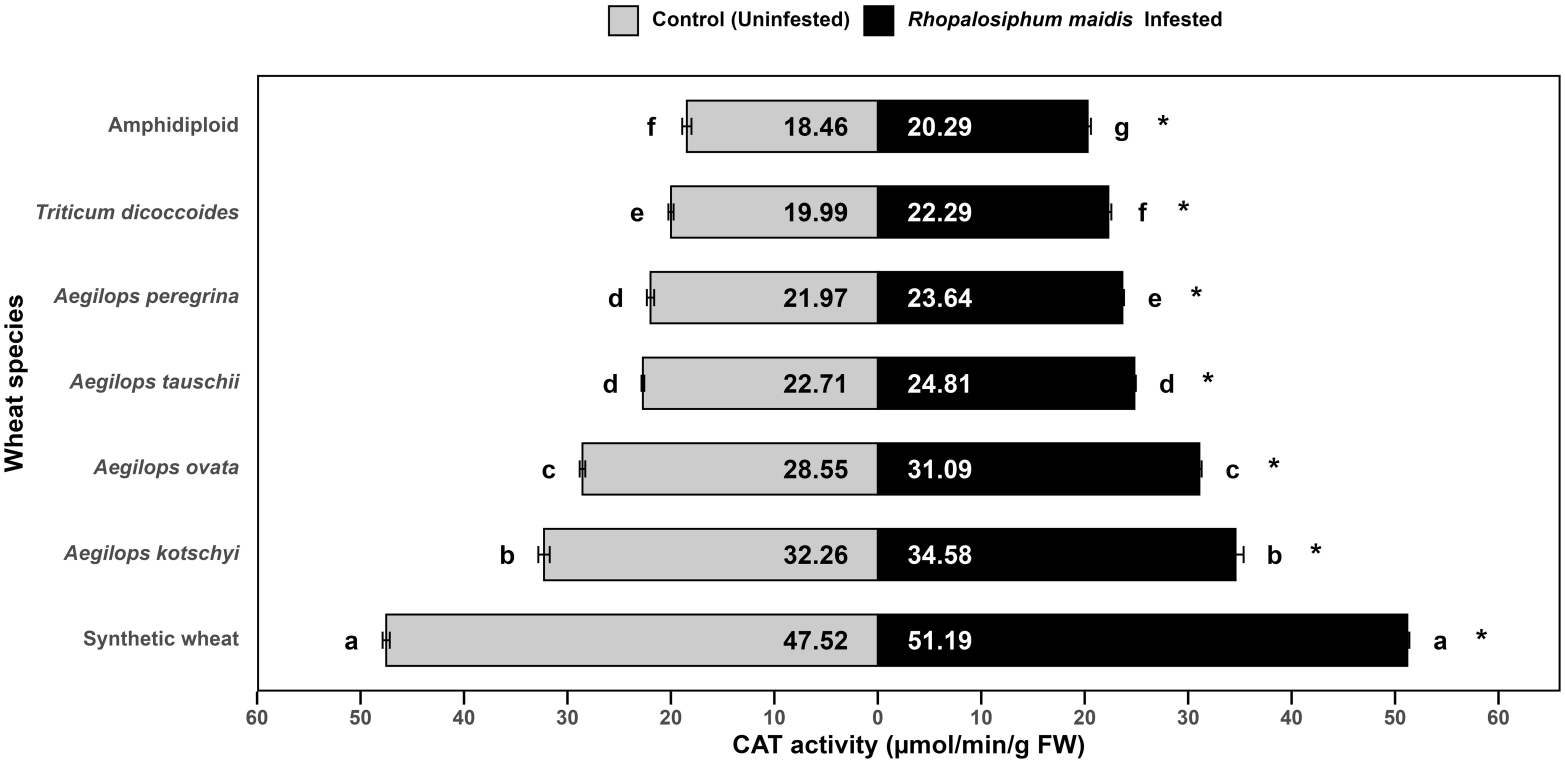
Mean CAT activity in the flag leaves of different wild and synthetic wheat species under *Rhopalosiphum maidis*-infested and uninfested (control) conditions. The lowercase letters depict significant differences among the mean CAT activity of different wheat species separately under *R. maidis*-infested and control (uninfested) conditions, with species sharing the same letter not significantly different as per Tukey's HSD test. However, the "*" in front of each wheat species row represents significant differences between CAT activity, according to pairwise comparisons using Sidak's adjustment in case of *R. maidis*-infested and control (uninfested) conditions for each wheat species separately.

The log-transformed mean CAT activity in the flag leaves of different wild and synthetic wheat species varied significantly under *R. maidis*-infested (*F*_6, 14_ = 2038, *p* < 0.05) and uninfested (control) (*F*_6, 14_ = 1543, *p* < 0.05) conditions (**Figure 2**). Across all species, infestation with *R. maidis* led to a significant (*F*_1, 28_ = 417.01, *p* < 0.05) increase in CAT activity indicated by "*" on the *R. maidis*-infested side in **Figure 2**. The magnitude of induction, however, differed among species. Among the tested species, synthetic wheat exhibited the highest mean CAT activity, recording 47.52 ± 0.34 µmol/min/g FW under control conditions and increasing further to 51.19 ± 0.19 µmol/min/g FW under aphid infestation. This was significantly higher than all the other species. *Aegilops kotschyi* and *Ae. ovata* showed comparatively high CAT activity (32.26 ± 0.55 and 28.55 ± 0.27 µmol/min/g FW under control and 34.58 ± 0.77 and 31.09 ± 0.21 µmol/min/g FW under *R. maidis* infestation, respectively), both differing significantly from the lower CAT activity of other species. Intermediate CAT levels were observed in *Ae. tauschii* and *Ae. peregrina*, with values of 22.71 ± 0.13 and 21.97 ± 0.35 µmol/min/g FW without infestation (control) and 24.81 ± 0.13 and 23.64 ± 0.15 µmol/min/g FW with aphid infestation, respectively. Meanwhile, *T. dicoccoides* showed slightly lower CAT activity (19.99 ± 0.25 in control and 22.20 ± 0.28 µmol/min/g FW in aphid-infested flag leaves). The lowest CAT activity was recorded in amphidiploid wheat, with 18.46 ± 0.44 (control) and 20.29 ± 0.31 µmol/min/g FW (infested), significantly lower than all the other species groups. The statistical groupings revealed that under both control and infested conditions, species differed significantly in CAT activity, with synthetic wheat clearly separated as the highest and amphidiploid wheat as the lowest. It is also confirmed from the observation that aphid infestation universally induced CAT activity, though the degree of induction varied depending on the wheat species. These findings suggest that the antioxidant defense response, as reflected by CAT activity, is differentially regulated among wheat species. The higher induction in synthetic wheat, *Ae. kotschyi*, and *Ae. ovata* indicates their stronger oxidative stress tolerance mechanisms under aphid attack compared to amphidiploid and *T. dicoccoides*, which showed the weakest responses.

### Ascorbate peroxidase activity response to *Rhopalosiphum maidis* infestation

**Figure 3:**
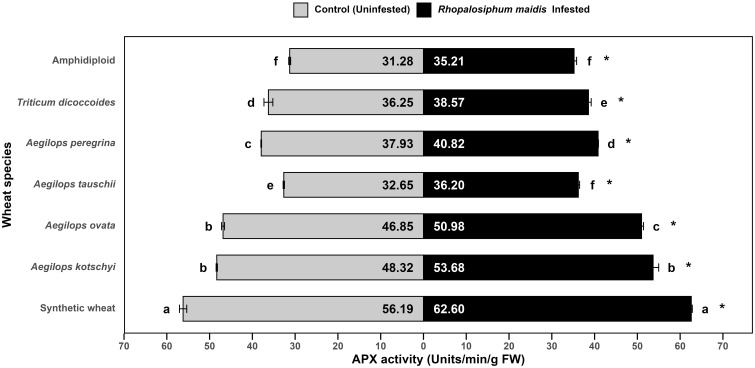
Mean APX activity in the flag leaves of different wild and synthetic wheat species under *Rhopalosiphum maidis*-infested and uninfested (control) conditions. The lowercase letters depict significant differences among the mean APX activity of different wheat species separately under *R. maidis*-infested and control (uninfested) conditions, with species sharing the same letter not significantly different as per Tukey's HSD test. However, the "*" in front of each wheat species row represents significant differences between APX activity, according to pairwise comparisons using Sidak's adjustment in case of *R. maidis*-infested and control (uninfested) conditions for each wheat species separately.

The log-transformed mean APX activity in the flag leaves of different wheat species showed significant variation under *R. maidis*-infested (*F*_6, 14_ = 793.2, *p* < 0.05) and uninfested (control) (*F*_6, 14_ = 841.2, *p* < 0.05) conditions (**Figure 3**). Across all species, *R. maidis* infestation significantly (*F*_1, 28_ = 512.94, *p* < 0.05) enhanced APX activity indicated by "*" on the *R. maidis*-infested side in **Figure 3**. However, the magnitude of this induction varied among species. Among the tested materials, synthetic wheat recorded the highest APX activity, with 56.19 ± 0.85 units/min/g FW under control and 62.60 ± 0.24 units/min/g FW under *R. maidis* infestation, significantly higher than all the other species. *Aegilops kotschyi* and *Ae. ovata* also exhibited high APX activities, which increased on aphid infestation from 48.32 ± 0.15 to 53.68 ± 1.30 units/min/g FW and from 46.85 ± 0.34 to 50.98 ± 0.45 units/min/g FW, respectively. Intermediate APX activity was observed in *Ae. peregrina* (from 37.93 ± 0.08 in the controlled condition to 40.82 ± 0.08 units/min/g FW under aphid-infested condition) and *T. dicoccoides* (from 36.25 ± 1.04 in the controlled condition to 38.57 ± 0.63 units/min/g FW under aphid-infested condition). Meanwhile, *Ae. tauschii* exhibited slightly lower APX activity, which under *R. maidis* infestation increased from 32.65 ± 0.15 to 36.20 ± 0.30 units/min/g FW. The lowest APX activity was found in amphidiploid wheat, with 31.28 ± 0.22 units/min/g FW under control and 35.21 ± 0.59 units/min/g FW under infestation. The statistical comparisons showed that synthetic wheat maintained significantly higher APX activity than all the other species, while amphidiploid consistently recorded the lowest levels. It is also indicated that aphid infestation universally upregulated APX activity, though the degree of increase was species dependent. These findings suggest that enhanced APX activity is a key oxidative stress response to *R. maidis* infestation, with synthetic wheat, *Ae. kotschyi*, and *Ae. ovata* displaying stronger antioxidant defense mechanisms compared to amphidiploid and *Ae. tauschii*, which showed the weakest responses.

### Peroxidase activity response to *Rhopalosiphum maidis* infestation

**Figure 4:**
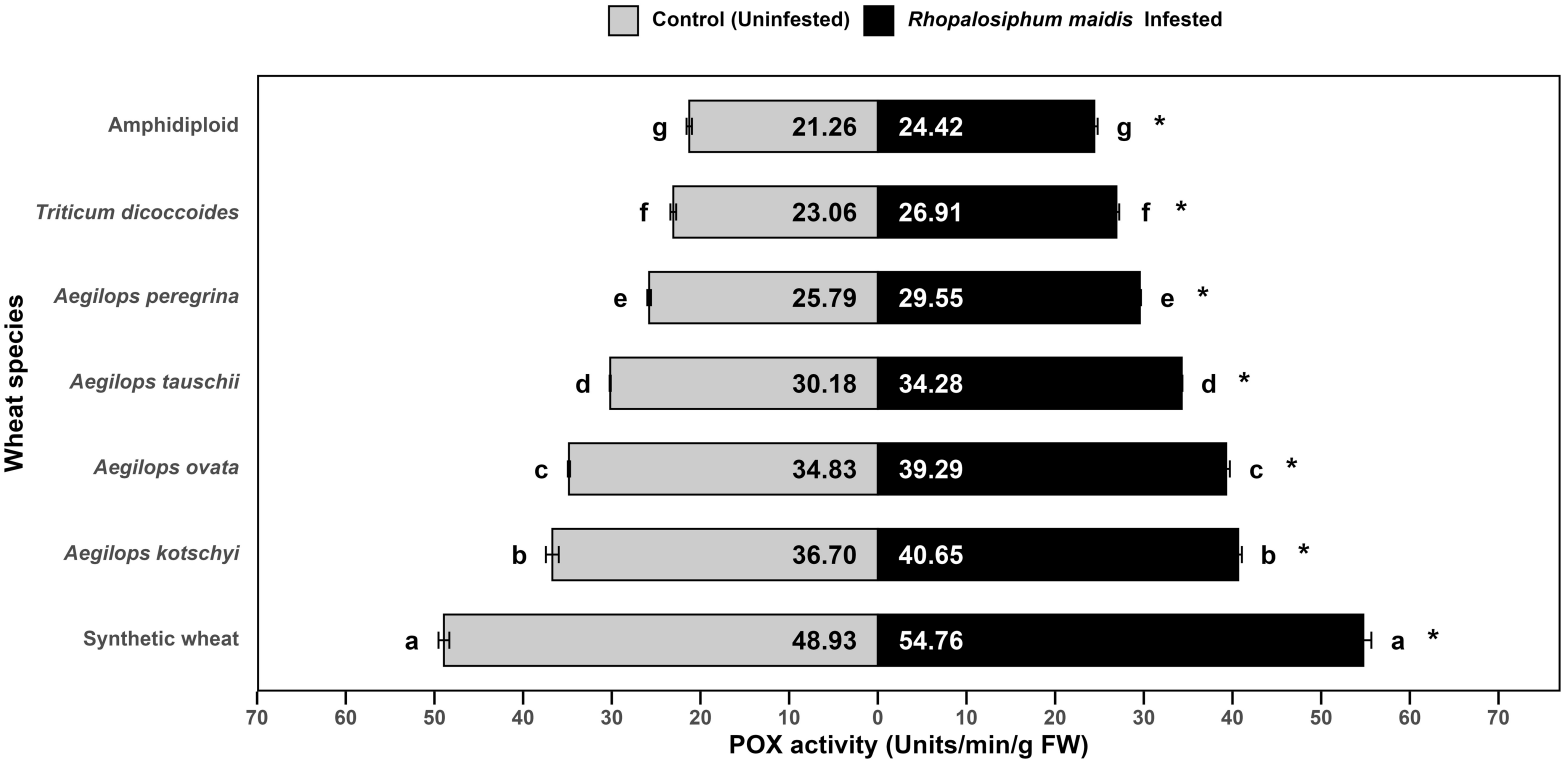
Mean POX activity in the flag leaves of different wild and synthetic wheat species under *R. maidis*-infested and uninfested (control) conditions. The lowercase letters depict significant differences among the mean POX activity of different wheat species separately under *R. maidis*-infested and control (uninfested) conditions, with species sharing the same letter not significantly different as per Tukey's HSD test. However, the "*" in front of each wheat species row represents significant differences between POX activity, according to pairwise comparisons using Sidak's adjustment in case of *R. maidis*-infested and control (uninfested) conditions for each wheat species separately.

The log-transformed mean POX activity in the flag leaves of wild and synthetic wheat species showed significant variation under *R. maidis*-infested (*F*_6, 14_ = 1821, *p* < 0.05) and uninfested (control) (*F*_6, 14_ = 1731, *p* < 0.05) conditions (**Figure 4**). Across all species, infestation with *R. maidis* significantly (*F*_1, 28_ = 1239.49, *p* < 0.05) increased POX activity for each species indicated by "*" on the *R. maidis*-infested side in **Figure 4**. However, the basal levels and the magnitude of induction differed considerably among species. Synthetic wheat exhibited the highest POX activity, with 48.93 ± units/min/g FW under control and 54.76 ± units/min/g FW under infestation, significantly higher than all the other species. *Aegilops kotschyi* and *Ae. ovata* also showed comparatively high activities (increasing from 36.70 ± 0.72 to 40.65 ± 0.41 and 34.83 ± 0.12 to 39.29 ± 0.41 units/min/g FW, respectively, upon aphid infestation) as compared to other tested species. Moderate activity was recorded in *Ae. tauschii* and *Ae. peregrina*, which also increased after aphid infestation from 30.18 ± 0.06 to 34.28 ± 0.07 units/min/g FW and from 25.79 ± 0.20 to 29.55 ± 0.13 units/min/g FW, respectively, while *T. dicoccoides* displayed relatively lower POX activity (which also significantly increased from 23.06 ± 0.32 to 26.91 ± 0.31 units/min/g FW). The lowest activity was observed in amphidiploid wheat, which showed 21.26 ± 0.30 units/min/g FW under control and 24.42 ± 0.37 units/min/g FW under infestation. The statistical comparisons revealed that synthetic wheat was distinctly superior in POX activity, while amphidiploid consistently showed the lowest activity. The aphid infestation significantly induced POX activity across all species, but the degree of induction was strongly species dependent. These results highlight that synthetic wheat, *Ae. kotschyi*, and *Ae. ovata* possess stronger peroxidase-based antioxidant defenses, potentially conferring greater tolerance to *R. maidis* infestation. In contrast, amphidiploid and *T. dicoccoides* showed weaker responses, suggesting limited oxidative stress tolerance.

### Glutathione reductase activity response to *Rhopalosiphum maidis* infestation

**Figure 5:**
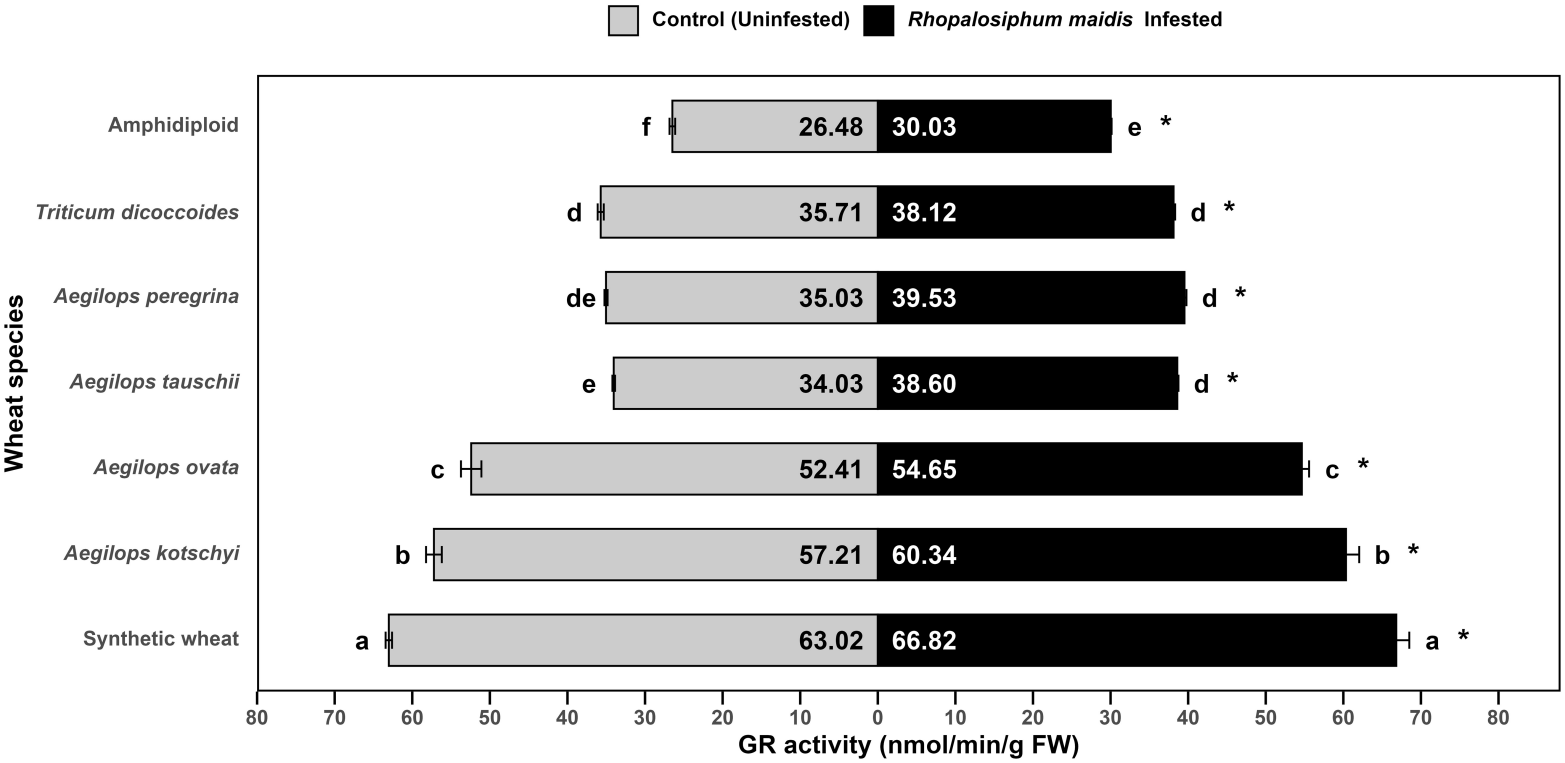
Mean GR activity in the flag leaves of different wild and synthetic wheat species under *Rhopalosiphum maidis*-infested and uninfested (control) conditions. The lowercase letters depict significant differences among the mean GR activity of different wheat species separately under *R. maidis*-infested and control (uninfested) conditions, with species sharing the same letter not significantly different as per Tukey's HSD test. However, the "*" in front of each wheat species row represents significant differences between GR activity, according to pairwise comparisons using Sidak's adjustment in case of *R. maidis*-infested and control (uninfested) conditions for each wheat species separately.

The log-transformed mean GR activity in the flag leaves of wheat species showed significant variation under *R. maidis*-infested (*F*_6, 14_ = 983.1, *p* < 0.05) and uninfested (control) (*F*_6, 14_ = 1,622, *p* < 0.05) conditions (**Figure 5**). In all species, aphid infestation led to a significant (*F*_1, 28_ = 331.63, *p* < 0.05) increase in GR activity indicated by "*" on the *R. maidis*-infested side in **Figure 5**. However, the basal levels as well as the magnitude of induction varied among species. Synthetic wheat recorded the highest GR activity, with 63.02 ± 0.41 nmol of NADP^+^ formed/min/g FW under control and 66.82 ± 1.67 under infested conditions, significantly higher than all the other species. *Aegilops kotschyi* also showed high GR activity with increased levels after aphid infestation from 57.21 ± 1.01 to 60.34 ± 1.70 nmol/min/g FW, followed by *Ae. ovata*, which increased from 52.41 ± 1.31 to 54.65 ± 0.93 nmol/min/g FW. Moderate GR activity was observed in *Ae. tauschii*, *Ae. peregrina*, and *T. dicoccoides*, which significantly increased under *R. maidis* infestation from 34.03 ± 0.17 to 38.60 ± 0.15 nmol/min/g FW, from 35.03 ± 0.20 to 39.53 ± 0.25, and from 35.71 ± 0.39 to 38.12 ± 0.20, respectively. The lowest GR activity was detected in amphidiploid wheat, with 26.48 ± 0.36 nmol/min/g FW in control and 30.03 ± 0.10 under infested conditions. The statistical comparisons showed that synthetic wheat consistently maintained the highest GR activity, significantly separating it from other species, while amphidiploid wheat exhibited the weakest activity. It was observed that *R. maidis* infestation induced GR activity in all species groups, though the degree of induction was highly dependent on the species. These findings indicate that synthetic wheat, *Ae. kotschyi*, and *Ae. ovata* possess robust glutathione reductase-mediated antioxidant responses, which may contribute to enhanced tolerance against *R. maidis*. In contrast, amphidiploid and *T. dicoccoides* appear less efficient in activating GR-based defense mechanisms.

### Phenylalanine ammonia-lyase activity response to *Rhopalosiphum maidis* infestation

**Figure 6:**
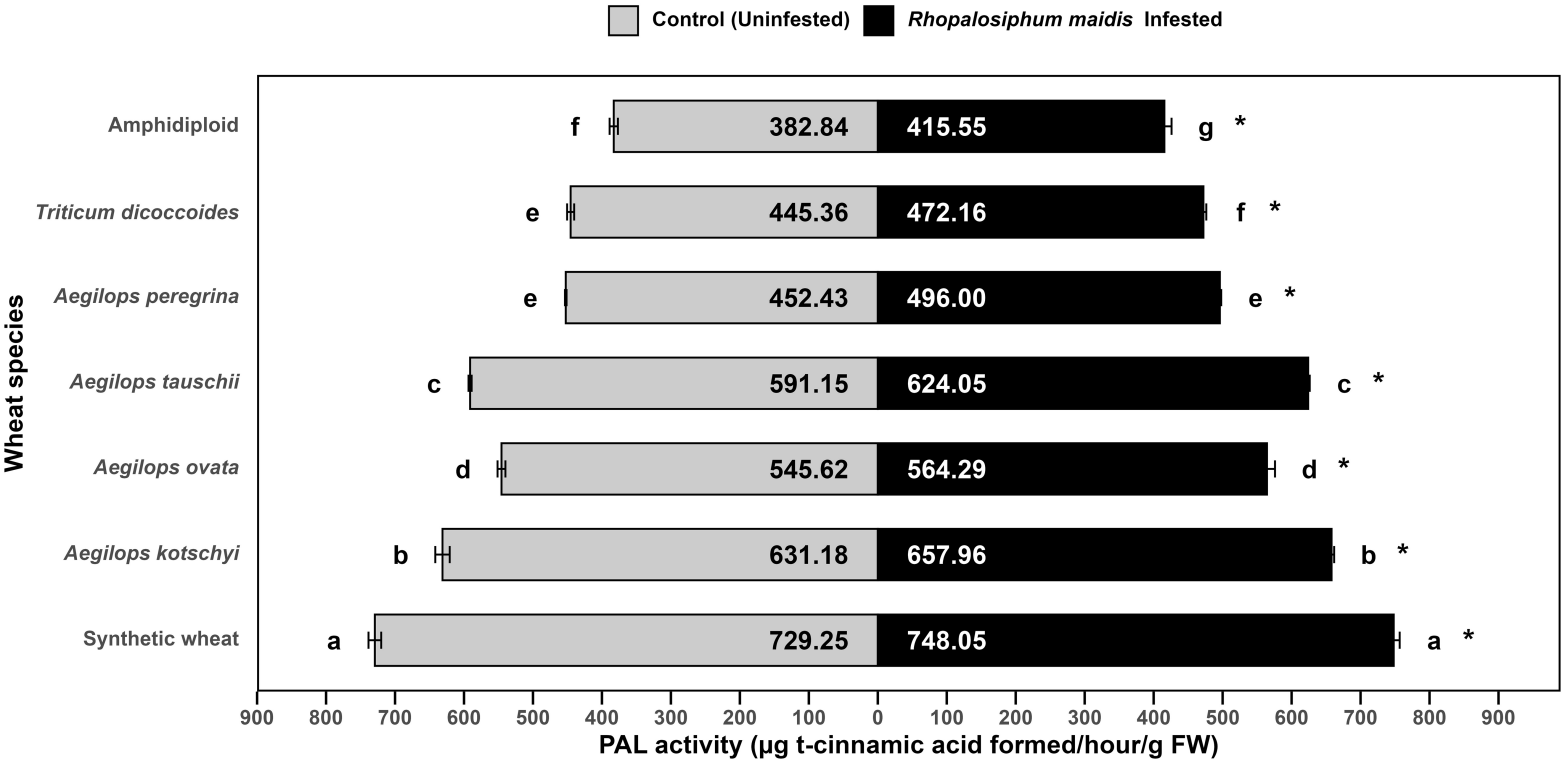
Mean PAL activity in the flag leaves of different wild and synthetic wheat species under *Rhopalosiphum maidis*-infested and uninfested (control) conditions. The lowercase letters depict significant differences among the mean PAL activity of different wheat species separately under *R. maidis*-infested and control (uninfested) conditions, with species sharing the same letter not significantly different as per Tukey's HSD test. However, the "*" in front of each wheat species row represents significant differences between PAL activity, according to pairwise comparisons using Sidak's adjustment in case of *R. maidis*-infested and control (uninfested) conditions for each wheat species separately.

The mean PAL activity in the flag leaves of wild and synthetic wheat species showed significant variation under *R. maidis*-infested (*F*_6, 14_ = 794.7, *p* < 0.05) and uninfested (control) (*F*_6, 14_ = 1,041, *p* < 0.05) conditions (**Figure 6**). Across all species, *R. maidis* infestation significantly (*F*_1, 28_ = 183.87, *p* < 0.05) enhanced PAL activity for each species indicated by "*" on the *R. maidis*-infested side in **Figure 6**. However, large differences were observed in the basal levels and extent of induction among the species. Synthetic wheat recorded the highest PAL activity, with 729.25 ± 9.21 µg t-cinnamic acid formed/h/g FW under control conditions and 748.05 ± 8.66 under infestation, significantly higher than all the other species. *Aegilops kotschyi* also exhibited high PAL activity (which increased from 631.18 ± 10.47 to 657.96 ± 3.72 µg t-cinnamic acid formed/h/g FW due to aphid infestation), followed by *Ae. tauschii* (which increased from 591.15 ± 2.26 to 624.05 ± 2.25 µg t-cinnamic acid formed/h/g FW due to aphid infestation) and *Ae. ovata* (which increased from 545.62 ± 5.70 to 564.29 ± 11.63 µg t-cinnamic acid formed/h/g FW due to aphid infestation). Moderate PAL activity was observed in *Ae. peregrina* (which significantly increased due to aphid infestation from 452.43 ± to 496.00 ± µg t-cinnamic acid formed/h/g FW) and *T. dicoccoides* (significantly increasing from 445.36 ± 5.14 to 472.16 ± 4.13 µg t-cinnamic acid formed/h/g FW due to *R. maidis* infestation). The lowest activity was recorded in amphidiploid wheat, with 382.84 ± 6.01 µg t-cinnamic acid formed/h/g FW under control and 415.55 ± 10.37 µg t-cinnamic acid formed/h/g FW under infestation, respectively. The statistical comparisons indicated that synthetic wheat and *Ae. kotschyi* maintained significantly higher PAL activity than all the other species, while amphidiploid consistently recorded the lowest activity. The aphid infestation induced PAL activity across all species groups, though the degree of induction varied by species. These results highlight that synthetic wheat, *Ae. kotschyi*, and *Ae. tauschii* possess stronger phenylpropanoid pathway activation under *R. maidis* attack, suggesting their enhanced defensive capacity, while amphidiploid and *T. dicoccoides* remain comparatively weak responders.

### Polyphenol oxidase activity response to *Rhopalosiphum maidis* infestation

**Figure 7:**
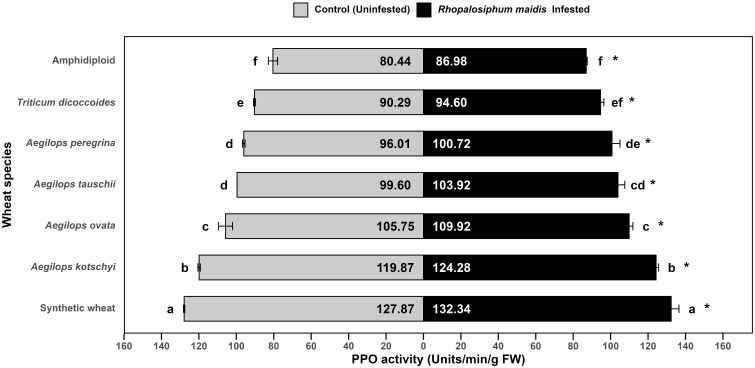
Mean PPO activity in the flag leaves of different wild and synthetic wheat species under *Rhopalosiphum maidis*-infested and uninfested (control) conditions. The lowercase letters depict significant differences among the mean PPO activity of different wheat species separately under *R. maidis*-infested and control (uninfested) conditions, with species sharing the same letter not significantly different as per Tukey's HSD test. However, the "*" in front of each wheat species row represents significant differences between PPO activity, according to pairwise comparisons using Sidak's adjustment in case of *R. maidis*-infested and control (uninfested) conditions for each wheat species separately.

The mean PPO activity in the flag leaves of wheat species showed significant variation under *R. maidis*-infested (*F*_6, 14_ = 93.28, *p* < 0.05) and uninfested (control) (*F*_6, 14_ = 271.7, *p* < 0.05) conditions (**Figure 7**). Across all species, aphid infestation significantly (*F*_1, 28_ = 41.00, *p* < 0.05) increased PPO activity indicated by "*" on the *R. maidis*-infested side in **Figure 7**. However, the basal PPO activities and magnitude of induction varied among species. Synthetic wheat recorded the highest PPO activity (127.87 ± 0.34 units/min/g FW under control and 132.34 ± 4.17 under infestation), significantly higher than all the other species. *Aegilops kotschyi* also exhibited high PPO activity (which increased from 119.87 ± 0.64 to 124.28 ± 1.34 units/min/g FW due to aphid infestation), followed by *Ae. ovata* (which significantly increased due to *R. maidis* infestation from 105.75 ± 3.71 to 109.92 ± 1.98 units/min/g FW). Moderate PPO activity was observed in *Ae. tauschii* and *Ae. peregrina* increasing from 99.60 ± 0.01 to 103.92 ± 3.65 units/min/g FW and from 96.01 ± 0.69 to 100.72 ± 4.26 units/min/g FW, respectively. Meanwhile, *T. dicoccoides* showed comparatively lower activity with 90.29 ± 0.48 units/min/g FW under the uninfested condition, which increased to 94.60 ± 1.79 units/min/g FW under aphid-infested conditions. The lowest PPO activity was found in amphidiploid wheat, with 80.44 ± 2.46 under control and 86.98 ± 0.55 units/min/g FW under infestation. The statistical grouping showed clear separation, with synthetic wheat and *Ae. kotschyi* exhibiting the strongest PPO response, while amphidiploid consistently displayed the lowest one. The *R. maidis* infestation induced PPO activity in all the species, though the strength of induction was species dependent. These findings suggest that synthetic wheat, *Ae. kotschyi*, and *Ae. ovata* mount stronger PPO-mediated biochemical defenses against *R. maidis*, while amphidiploid and *T. dicoccoides* display weaker defense responses.

### Principal component analysis

**Figure 8:**
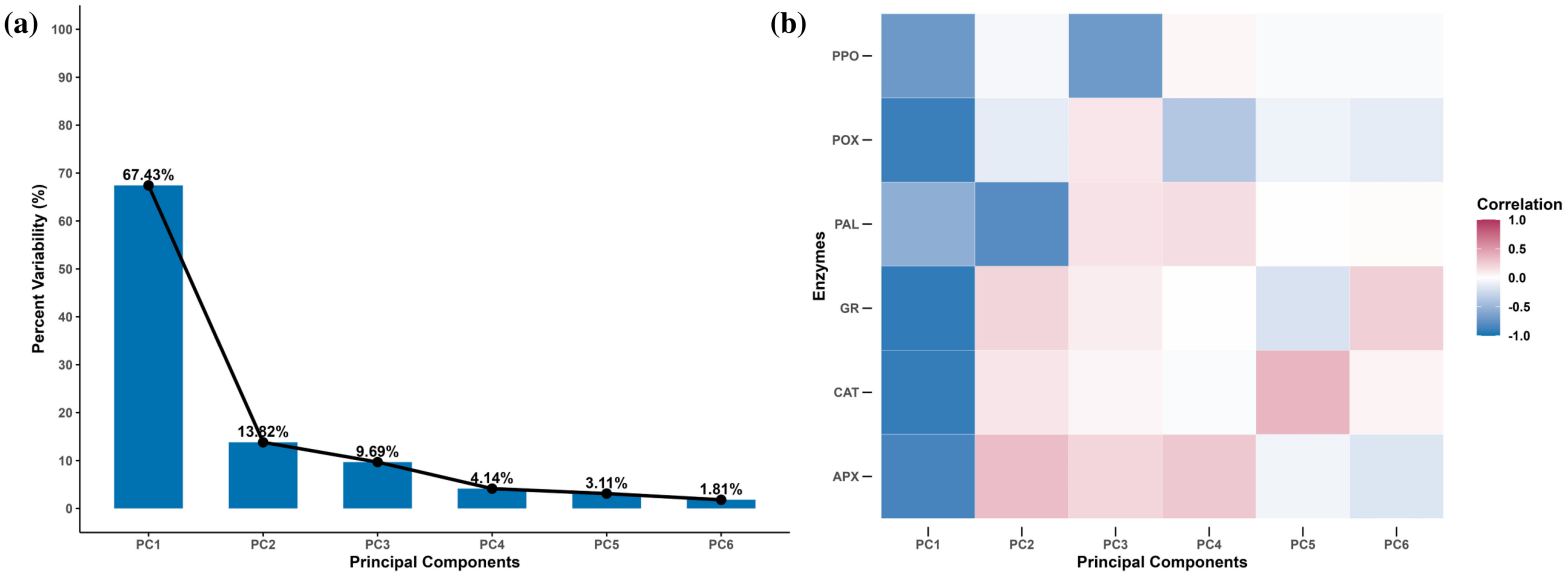
Scree plot **(A)** and correlation plot **(B)** from a principal component analysis on the antioxidant enzyme activity in different wheat species under *R. maidis*-infested and uninfested (control) conditions. The scree plot represents the variability explained by each principal component. The correlation plot depicts how each enzymatic activity is correlated with each dimension (principal component). The dark red color indicates a strong positive correlation, and the dark blue color indicates a strong negative correlation. A white square indicates the absence of correlation between an enzyme activity and a principal component.

**Figure 9:**
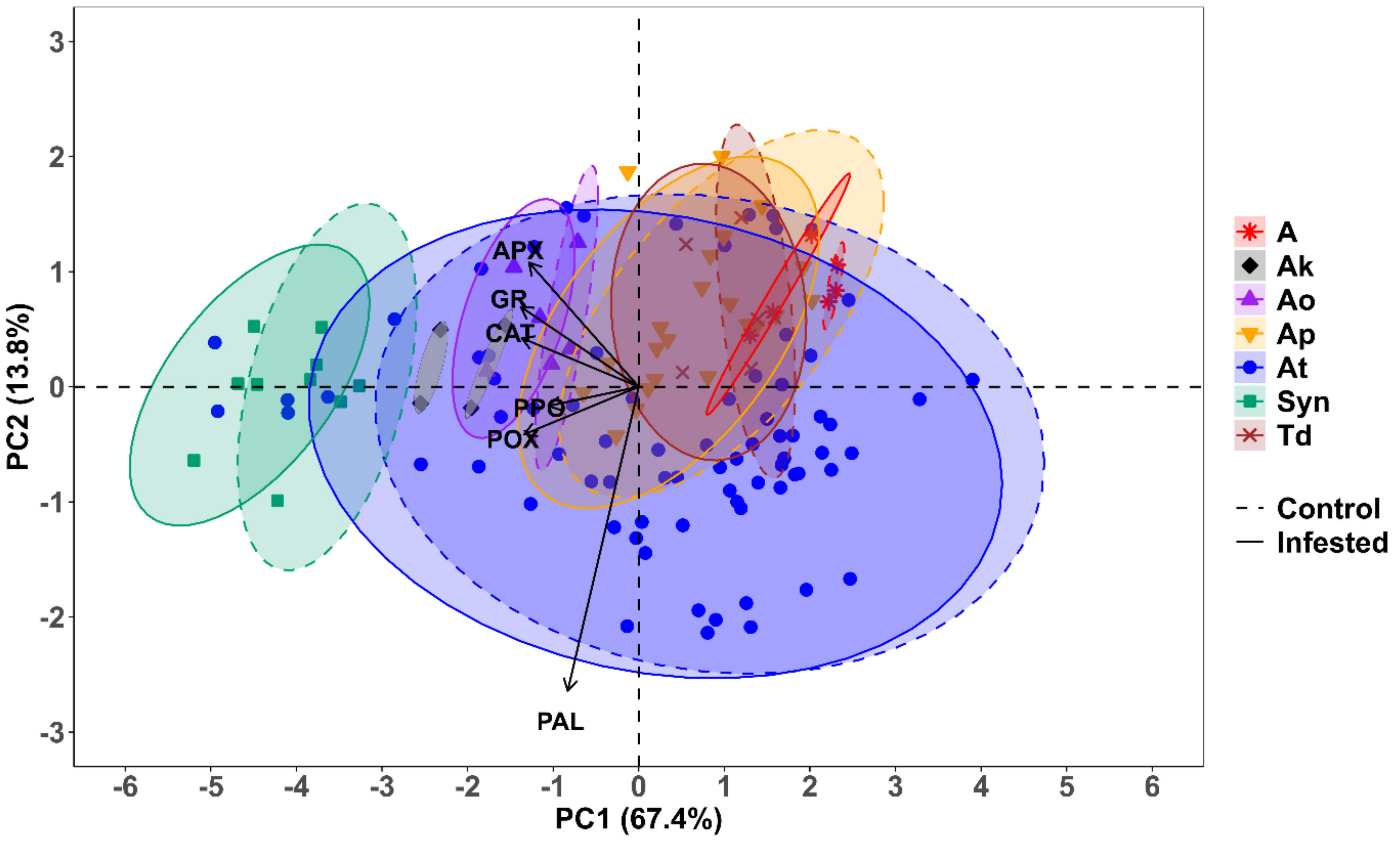
Principal component analysis biplot of the correlation between antioxidant enzyme activity and the first two principal components (PC1 and PC2) in different wheat species under *R. maidis*-infested and uninfested (control) conditions. The length and direction of the arrows indicate the strength of the correlation between the enzyme activity and the principal component. The dashed and solid ellipses represent the cluster for each wheat species under *R. maidis*-infested and uninfested (control) conditions, respectively.

Out of the six principal components (PCs), only one, i.e., PC1, had an eigenvalue of more than 1 and contributed 67.43% of the total cumulative variability among different genotypes (**Supplementary Table S2**). The PCA showed that principal component 1 (PC1) alone explained 67.43% of the variation, while principal components 2 (PC2) and 3 (PC3) explained 13.82% and 9.69%, respectively (**Figure 8A**). The enzymes Cat, APX, GR, and POX were strongly negatively correlated with PC1, while PAL and PPO were weakly negatively correlated with PC1 (**Figure 8B**). Variation in the PAL activity was mainly explained by PC2, while PC3 was moderately negatively correlated with PPO as well (**Figure 8B**). We decided to retain PC1 and PC2 for the subsequent analyses, as together they explained 81.25% of the total variation, and all variables were strongly represented by these two dimensions. The biplot depicted all wheat genotypes for the six enzymes. These two principal components were plotted as a biplot with PC1 on the *x*-axis and PC2 on the *y*-axis to detect the association between different clusters. In the PCA biplot, a vector was drawn from the origin to every enzyme, which enables the visualization of interrelationships among enzyme activities (**Figure 9**). Infested plants tend to group more with enzyme activity (right side), suggesting that infestation induces higher oxidative stress response. Moreover, the clusters for the wheat species are overlapping, but *Ae. tauschii* (At) has higher principal component scores on PC1 (**Figure 9**).

## Discussion

The results of the present investigation clearly demonstrate that wheat genotypes exhibit strong variability in their biochemical defense responses against *R. maidis*. The wild and synthetic wheat species exhibit differential biochemical responses to corn leaf aphid (*R. maidis*) infestation, as reflected in variations in antioxidant enzyme activities and aphid-induced mortality. The enhanced mortality of aphid nymphs in resistant genotypes such as amphidiploid wheat and *Ae. kotschyi*, coupled with the significant upregulation of antioxidant enzymes across all species, highlights the multifaceted nature of host–aphid interactions. These findings emphasize that wheat defense against *R. maidis* relies on a combination of constitutive resistance traits and inducible biochemical responses, with oxidative stress management and secondary metabolism activation emerging as central components, as reflected in the significant genotypic differences observed.

### Antioxidant enzyme responses as core defense mechanisms

The study confirmed that infestation by *R. maidis* consistently induced CAT, APX, POX, and GR across all species. This is in line with the well-established role of ROS as both signaling molecules and sources of oxidative stress during insect feeding (Tyagi et al., 2022). The induction of ROS-scavenging enzymes prevents oxidative damage to cell membranes and organelles, thereby preserving physiological processes such as photosynthesis and nutrient allocation (Akter et al., 2015). Similar increases in antioxidant enzyme activities have been reported in wheat challenged with *S. avenae* or wheat aphid complex (Zhang et al., 2022; Kaur et al., 2017) and in cotton under *A. gossypii* attack (Li JinBu et al., 2008), suggesting a conserved defense strategy across cereal and fiber crops.

Among the tested species, synthetic wheat consistently exhibited the highest activity levels of CAT, APX, POX, and GR under both control and infested conditions. This finding suggests that synthetic wheat possesses a more robust oxidative stress management system, which could be a consequence of its diverse genetic background. Synthetic hexaploid wheats are known to combine genomes from *T. turgidum* and *Ae. tauschii*, often conferring novel resistance traits, including tolerance to biotic and abiotic stresses (Chhuneja et al., 2016; Zaynab et al., 2018). These findings provide evidence that synthetic wheat lines could serve as valuable genetic donors for enhancing aphid resistance in elite cultivars.

Interestingly, amphidiploid wheat displayed the lowest antioxidant enzyme activity yet recorded the highest aphid mortality. This divergence suggests that amphidiploid resistance is likely mediated by mechanisms other than oxidative stress detoxification, possibly involving constitutive traits such as higher levels of phenolics, trichomes, or altered phloem composition. Similar non-enzymatic contributions to aphid resistance have been documented in barley, where PAL activity and phenolic accumulation were more closely correlated with resistance to *Rhopalosiphum padi* than antioxidant enzymes (Chaman et al., 2003). This fact indicates that multiple, and sometimes independent, resistance pathways are employed by wheat genotypes against aphids. Apart from the transcriptomics and metabolomics, proteomics can also provide insights into the mechanisms underlying the wheat resistance to aphids, as shown by the study conducted by Kosová et al. (2022), where the combined effect of water deficit and aphid infestation in two spring wheat varieties led to the induction of proteins involved in detoxification processes, by altering 113 proteins linked to signaling, energy metabolism, redox regulation, defense, and secondary metabolism, indicating long-term plant adaptation to stress.

### The phenylpropanoid pathway and secondary metabolism in defense

In addition to ROS-scavenging enzymes, the induction of PAL and PPO activities was particularly notable in resistant species such as synthetic wheat and *Ae. kotschyi*. Both enzymes are integral to the phenylpropanoid pathway, which produces a range of secondary metabolites including phenolic acids, flavonoids, and lignin. These compounds function as feeding deterrents, reinforce cell walls, and interfere with aphid digestion and fecundity (Dixon et al., 2002; Fürstenberg-Hägg et al., 2013).

The higher PAL and PPO activities observed in resistant lines of the present study are consistent with previous findings in wheat and cotton, where resistant cultivars exhibited stronger induction of these enzymes compared to susceptible ones (Han et al., 2009; Sha et al., 2015). For example, elevated PPO activity has been associated with reduced aphid population growth in sorghum (Chang et al., 2008) and with stronger lignification in rice under insect stress (Usha Rani and Jyothsna, 2010). Therefore, activation of the phenylpropanoid pathway appears to be a reliable biochemical marker for resistance against phloem-feeding insects, including *R. maidis*.

### Species-dependent resistance mechanisms

The variation in aphid-induced mortality across accessions of *Ae. tauschii* and *Ae. peregrina* further supports the role of genetic background in shaping biochemical responses. Some accessions exhibited high aphid mortality coupled with intermediate enzyme activities, suggesting that species-specific interactions determine the balance between constitutive and inducible defenses. The observed diversity in defense responses within a single species echoes the findings from Greenslade et al. (2016) and Naqvi et al. (2017), who demonstrated that even within closely related genotypes, multiple resistance mechanisms can operate simultaneously, ranging from basal immunity to induced responses.

The species-dependent differences observed here are also important from a breeding perspective. While synthetic wheat showed strong enzymatic responses, amphidiploid wheat offered high aphid mortality despite low enzyme activity. Combining these complementary traits through hybridization or marker-assisted selection could enhance the durability of resistance in cultivated wheat. Recent advances in genomics-assisted breeding now allow the precise mapping of resistance-related biochemical traits, thereby enabling the pyramiding of multiple defense mechanisms (Li et al., 2021).

### Implications for integrated pest management and breeding

From a crop protection standpoint, reliance on chemical insecticides against aphids has been increasingly challenged by resistance development and environmental concerns (Liu et al., 2018; Li et al., 2021). The identification of wheat genotypes with strong biochemical defense responses provides an eco-friendly alternative for sustainable aphid management. Resistant species such as synthetic wheat, *Ae. kotschyi*, and amphidiploid wheat could serve as parental lines in breeding programs aimed at developing cultivars that are less dependent on chemical control.

Furthermore, the universal induction of antioxidant enzymes in all species suggests that ROS signaling constitutes a conserved first line of defense. However, the magnitude of enzyme activity induction differentiates resistant and susceptible genotypes. These findings reinforce the need for screening wheat germplasm not only for morphological traits but also for dynamic biochemical responses under pest pressure. Future research integrating metabolomic and transcriptomic analyses could provide deeper insights into the regulatory networks underlying these biochemical defenses (Naqvi et al., 2017; Zhang et al., 2022; Jasrotia et al., 2022a).

## Conclusion

In conclusion, wheat resistance to *R. maidis* is mediated by a multifaceted defense strategy involving both oxidative stress management and phenylpropanoid metabolism, with clear variation across wheat species and their genotypes. The enhanced activities of antioxidant enzymes such as CAT, APX, POX, and GR highlight their central role in mitigating aphid-induced oxidative damage, while phenylpropanoid pathways contribute to the reinforcement of structural and chemical defenses. Synthetic wheat and *Ae. kotschyi* stand out as valuable genetic resources due to their robust enzymatic responses, offering strong potential for the incorporation of antioxidant-based resistance traits. In contrast, amphidiploid wheat, despite showing lower enzymatic induction, exerts significant aphid mortality, suggesting the presence of unique non-enzymatic or secondary metabolite-based defense mechanisms that are equally important in shaping resistance. Together, these findings underscore the importance of exploiting diverse genetic pools to capture complementary resistance strategies. The integration of enzymatic and non-enzymatic resistance factors through advanced breeding approaches, including marker-assisted and molecular breeding, provides a viable pathway for developing durable, broad-spectrum, and environmentally safe aphid-resistant wheat varieties. Such advancements will not only contribute to sustainable crop protection and reduced pesticide dependency but also enhance the resilience of wheat cultivation under changing pest dynamics and climate conditions.

## Data Availability

The original contributions presented in the study are included in the article/**Supplementary Material**. Further inquiries can be directed to the corresponding author.
