# Supplementary material for: Antioxidant enzyme responses in different wheat species infested with the corn leaf aphid, Rhopalosiphum maidis Fitch
Source: Front Plant Sci. 2025 Oct 31;16:1693782. doi: 10.3389/fpls.2025.1693782 (PMC12615455; doi:10.3389/fpls.2025.1693782)
Supplement: Supplementary file 1 [file Table1.doc]

Table S1. Mean CAT (catalase), APX (ascorbate peroxidase), POX (Peroxidase), GR (glutathione reductase), PAL (Phenylalanine ammonia lyase) and PPO (Polyphenol oxidase) activity in flag leaves of different accessions of wild and synthetic wheat species under *Rhopalosiphum maidis* infested and control (uninfested) conditions.

| **Wheat species** | **Accession** | **CAT activity (µmol/min/g FW)** | | **APX activity (Units/min/g FW)** | | **POX activity (Units/min/g FW)** | | **GR activity (nmol/min/g FW)** | | **PAL activity (µg t-cinnamic acid formed/hour/g FW)** | | **PPO activity (Units/min/g FW)** | |
| --- | --- | --- | --- | --- | --- | --- | --- | --- | --- | --- | --- | --- | --- |
|  |  | **Control** | ***R. maidis* Infested** | **Control** | ***R. maidis* Infested** | **Control** | ***R. maidis* Infested** | **Control** | ***R. maidis* Infested** | **Control** | ***R. maidis* Infested** | **Control** | ***R. maidis* Infested** |
| *Aegilops tauschii* | AetaNA1 | 26.71±0.15 | 27.49±0.22 | 40.02±0.34 | 41.68±0.59 | 32.64±0.19 | 37.86±0.83 | 50.45±0.24 | 52.25±0.46 | 632.78±14.23 | 638.43±3.92 | 114.43±9.25 | 118.64±1.04 |
|  | 13763a | 17.50±0.11 | 19.96±0.09 | 19.16±0.46 | 20.21±0.36 | 30.82±0.23 | 33.54±0.07 | 27.45±0.56 | 29.25±0.05 | 522.50±11.69 | 540.53±9.84 | 90.69±2.22 | 96.45±1.76 |
|  | 3761a | 25.50±0.08 | 29.40±0.28 | 31.26±0.62 | 33.81±0.74 | 25.78±0.19 | 27.30±0.52 | 42.21±0.07 | 47.76±0.32 | 785.64±12.26 | 807.39±14.70 | 91.13±10.72 | 96.61±2.52 |
|  | PI 554324 | 18.59±0.11 | 22.49±0.55 | 19.82±0.46 | 21.75±0.52 | 32.47±0.62 | 35.95±0.62 | 18.57±0.02 | 21.57±0.34 | 534.64±11.69 | 591.64±6.15 | 80.53±1.01 | 88.46±2.26 |
|  | 9787a | 25.93±0.23 | 28.03±0.06 | 46.57±0.73 | 55.81±0.99 | 46.71±0.02 | 53.52±0.11 | 59.02±1.35 | 65.88±0.85 | 614.72±15.03 | 653.64±6.46 | 112.90±19.35 | 115.61±0.71 |
|  | 12a | 19.46±0.46 | 21.41±0.47 | 21.24±0.24 | 22.54±0.09 | 27.04±0.13 | 29.83±0.47 | 19.37±0.04 | 22.37±0.41 | 398.92±6.64 | 434.39±5.65 | 101.14±4.74 | 111.89±0.47 |
|  | 14578a | 17.12±0.38 | 20.78±0.31 | 20.08±0.19 | 21.66±0.06 | 32.99±0.43 | 36.42±0.24 | 18.21±0.11 | 20.21±0.41 | 777.93±8.50 | 797.89±1.67 | 98.51±2.05 | 101.26±1.95 |
|  | 3761b | 16.02±0.19 | 18.84±0.16 | 34.24±0.46 | 35.02±0.38 | 19.29±0.17 | 21.65±0.35 | 22.41±0.52 | 23.92±0.38 | 406.82±6.77 | 457.50±11.42 | 79.87±1.12 | 94.61±0.09 |
|  | 1 | 20.39±0.20 | 23.82±0.25 | 30.82±0.48 | 32.42±0.46 | 35.62±0.78 | 40.21±0.84 | 21.08±0.25 | 23.07±0.24 | 670.03±12.55 | 700.78±7.29 | 83.44±0.04 | 91.14±0.09 |
|  | 13764b | 23.71±0.18 | 24.41±0.24 | 21.60±0.06 | 23.27±0.39 | 28.55±0.43 | 30.32±0.32 | 31.13±0.05 | 34.12±0.74 | 244.35±4.06 | 274.64±4.29 | 105.24±7.83 | 117.90±0.06 |
|  | 14578b | 16.88±0.15 | 18.25±0.28 | 20.48±0.07 | 22.05±0.12 | 37.91±0.97 | 41.99±0.31 | 15.92±0.29 | 21.76±0.52 | 727.35±14.01 | 752.32±3.52 | 98.94±0.00 | 101.99±1.54 |
|  | 9829b | 9.26±0.14 | 10.95±0.22 | 17.27±0.13 | 20.83±0.44 | 21.42±0.16 | 23.64±0.49 | 16.04±0.26 | 19.53±0.48 | 696.18±10.51 | 735.04±5.74 | 95.92±0.25 | 98.58±0.10 |
|  | 62a | 11.23±0.12 | 12.71±0.09 | 24.54±0.07 | 27.04±0.35 | 18.74±0.34 | 22.05±0.48 | 22.10±0.21 | 27.41±0.15 | 521.25±2.98 | 557.03±8.12 | 102.87±1.18 | 106.79±2.56 |
|  | 9803a | 16.57±0.18 | 18.67±0.37 | 28.41±0.13 | 33.68±0.21 | 15.37±0.35 | 18.65±0.35 | 17.39±0.19 | 18.35±0.12 | 602.43±14.64 | 654.32±6.19 | 82.77±2.01 | 87.29±2.10 |
|  | 14325a | 20.89±0.19 | 22.73±0.22 | 26.72±0.46 | 29.87±0.08 | 27.50±0.29 | 31.01±0.15 | 23.43±0.08 | 25.43±0.17 | 585.93±11.28 | 621.82±15.85 | 97.40±0.45 | 106.94±0.97 |
|  | 2a | 24.33±0.33 | 26.01±0.08 | 28.76±0.03 | 32.38±0.14 | 23.22±0.29 | 24.97±0.44 | 37.84±0.06 | 42.94±0.71 | 286.78±7.31 | 319.57±1.66 | 105.27±13.29 | 121.413±2.97 |
|  | 3744b | 25.77±0.44 | 27.49±0.20 | 47.35±0.96 | 50.25±1.02 | 28.19±0.72 | 30.14±0.74 | 44.04±0.92 | 49.37±1.00 | 722.35±9.02 | 765.92±6.38 | 98.42±7.13 | 101.23±3.14 |
|  | 14336a | 24.64±0.11 | 28.97±0.08 | 28.05±0.10 | 35.53±0.61 | 24.91±0.26 | 27.75±0.52 | 39.41±0.53 | 43.92±0.41 | 781.64±6.10 | 803.25±7.52 | 83.56±10.12 | 85.73±2.29 |
|  | 59b | 16.06±0.28 | 18.01±0.34 | 21.63±0.03 | 26.21±0.37 | 22.03±0.19 | 25.61±0.63 | 39.35±0.57 | 41.35±0.40 | 498.57±7.78 | 540.28±5.34 | 97.38±1.32 | 102.04±2.34 |
|  | 14325b | 54.93±1.37 | 56.93±0.47 | 67.37±0.63 | 70.03±1.61 | 52.26±0.98 | 61.53±1.57 | 65.10±0.71 | 70.98±0.03 | 838.93±3.93 | 845.71±3.52 | 100.34±7.77 | 104.44±2.00 |
|  | 14336b | 24.28±0.34 | 26.72±0.58 | 37.97±0.14 | 42.86±0.04 | 30.42±0.51 | 32.87±0.84 | 46.77±0.12 | 55.88±0.93 | 611.68±7.64 | 662.11±0.68 | 102.01±16.96 | 106.46±1.279 |
|  | 3784a | 46.40±0.46 | 49.44±1.03 | 57.00±0.03 | 65.61±0.95 | 48.39±0.68 | 54.94±0.60 | 61.76±0.13 | 67.25±0.84 | 629.85±2.29 | 679.39±14.14 | 104.03±3.75 | 108.23±2.15 |
|  | 3757a | 15.09±0.28 | 16.57±0.32 | 18.96±0.09 | 24.71±0.48 | 28.48±0.29 | 29.92±0.64 | 15.59±0.18 | 20.39±0.07 | 541.03±4.22 | 561.07±7.88 | 93.13±2.33 | 96.01±1.65 |
|  | 62b | 25.07±0.07 | 27.77±0.56 | 26.85±0.66 | 29.16±0.73 | 43.18±0.49 | 46.96±0.48 | 41.75±0.93 | 45.49±0.64 | 634.42±3.29 | 681.32±0.71 | 118.82±7.83 | 126.08±2.56 |
|  | 9822a | 14.39±0.02 | 15.17±0.04 | 25.92±0.24 | 27.48±0.16 | 26.01±0.04 | 27.86±0.62 | 26.19±0.11 | 30.59±0.25 | 628.89±1.64 | 661.61±12.74 | 94.46±1.38 | 101.20±2.11 |
|  | 3757b | 21.80±0.30 | 24.72±0.13 | 36.85±0.15 | 40.74±0.99 | 20.51±0.17 | 22.38±0.16 | 24.21±0.32 | 26.87±0.65 | 325.35±1.69 | 330.71±4.82 | 85.91±1.43 | 87.78±2.24 |
|  | 62c | 27.49±0.49 | 28.73±0.09 | 43.67±0.05 | 52.87±1.34 | 39.55±0.85 | 44.32±0.03 | 53.31±0.29 | 59.61±1.11 | 585.57±13.31 | 628.07±8.92 | 95.21±3.38 | 104.38±1.29 |
|  | 3806a | 28.70±0.25 | 29.75±0.46 | 49.09±0.28 | 53.79±0.50 | 44.07±1.03 | 47.02±0.42 | 57.45±0.21 | 63.13±1.45 | 443.93±1.38 | 507.78±10.31 | 104.53±5.58 | 105.54±2.52 |
|  | 9830 | 21.21±0.20 | 23.86±0.07 | 27.79±0.19 | 31.48±0.26 | 16.89±0.19 | 24.31±0.34 | 21.39±0.53 | 33.13±0.12 | 622.57±13.93 | 670.39±6.97 | 100.70±8.33 | 111.88±0.29 |
|  | 8b | 24.98±0.17 | 26.99±0.11 | 52.56±1.04 | 55.41±0.87 | 34.66±0.74 | 36.66±0.56 | 48.86±0.08 | 57.25±0.09 | 399.75±6.45 | 431.75±6.73 | 96.57±15.19 | 106.88±2.37 |
|  | 13765 | 20.96±0.05 | 22.51±0.14 | 37.31±0.54 | 41.01±0.98 | 26.58±0.64 | 28.58±0.56 | 28.45±0.16 | 37.84±0.37 | 672.21±14.69 | 717.36±17.17 | 116.73±0.00 | 117.85±0.25 |
|  | 3806b | 13.57±0.06 | 15.48±0.21 | 24.94±0.36 | 28.31±0.03 | 21.59±0.51 | 24.59±0.35 | 21.64±0.26 | 25.64±0.13 | 511.74±2.39 | 534.50±1.94 | 99.47±0.72 | 106.12±2.43 |
|  | 3 | 24.06±0.46 | 25.65±0.44 | 23.25±0.40 | 26.87±0.53 | 26.44±0.14 | 33.09±0.62 | 35.13±0.84 | 39.41±0.65 | 587.32±1.83 | 612.39±1.58 | 108.64±5.55 | 111.41±0.49 |
|  | 18 | 22.46±0.44 | 24.52±0.21 | 17.61±0.01 | 21.41±0.49 | 31.27±0.23 | 35.89±0.73 | 30.45±0.08 | 36.66±0.53 | 803.36±5.01 | 829.64±6.47 | 95.65±6.76 | 112.89±2.55 |
|  | 16 | 51.08±1.17 | 53.03±0.74 | 61.07±0.13 | 63.88±1.47 | 50.04±0.02 | 59.58±0.84 | 63.53±0.09 | 68.82±0.07 | 773.93±12.48 | 796.28±8.71 | 96.77±8.01 | 105.24±1.69 |
|  | 15a | 27.99±0.69 | 29.09±0.59 | 54.79±0.91 | 56.79±0.56 | 41.41±0.86 | 52.50±0.25 | 55.49±0.55 | 61.96±0.74 | 795.89±12.83 | 798.64±3.33 | 100.88±3.72 | 102.07±0.98 |
|  | 13763b | 12.28±0.18 | 13.73±0.28 | 33.31±0.38 | 38.57±0.28 | 22.64±0.34 | 29.30±0.54 | 16.39±0.04 | 18.76±0.42 | 618.96±5.47 | 658.93±15.43 | 99.33±0.93 | 104.33±0.87 |
|  | 12b | 9.55±0.22 | 11.58±0.28 | 16.52±0.11 | 18.52±0.34 | 11.18±0.02 | 17.83±0.06 | 14.39±0.04 | 16.57±0.13 | 427.64±3.78 | 459.75±6.45 | 76.32±1.63 | 79.69±0.46 |
| Synthetic wheat | SYN9 | 50.30±0.22 | 51.86±0.91 | 54.00±0.37 | 60.05±1.22 | 44.07±0.00 | 47.02±0.96 | 58.72±1.51 | 61.90±1.41 | 712.93±17.88 | 735.46±0.69 | 122.77±1.39 | 125.23±0.00 |
|  | SYN5 | 53.03±0.14 | 56.05±0.88 | 53.86±0.81 | 58.52±0.21 | 48.84±0.08 | 55.49±0.49 | 63.84±0.56 | 69.59±0.65 | 630.53±7.54 | 656.78±10.25 | 128.64±2.88 | 130.09±0.88 |
|  | SYN74 | 41.60±0.59 | 44.72±1.12 | 59.11±1.11 | 66.11±1.07 | 54.03±1.32 | 62.06±0.84 | 67.88±0.31 | 72.03±1.16 | 884.47±9.21 | 851.42±10.63 | 130.44±2.51 | 140.38±3.22 |
|  | SYN20 | 45.09±0.77 | 48.19±1.15 | 56.13±0.56 | 63.05±0.07 | 51.35±0.32 | 58.01±0.69 | 65.80±1.16 | 68.06±0.92 | 675.75±1.76 | 731.43±11.03 | 132.21±3.09 | 137.58±0.65 |
|  | SYN87 | 47.57±0.01 | 55.09±0.06 | 57.86±0.54 | 65.27±1.19 | 46.34±0.19 | 51.21±0.27 | 58.84±1.34 | 62.53±1.33 | 742.57±1.93 | 765.14±10.75 | 125.29±1.11 | 128.42±1.54 |
| *Aegilops kotschyi* | 3774a | 31.31±0.65 | 33.41±0.64 | 49.93±0.69 | 55.28±0.76 | 35.14±0.84 | 38.91±0.43 | 55.70±0.95 | 59.12±1.20 | 574.64±4.81 | 606.64±0.27 | 119.82±1.02 | 127.62±1.08 |
|  | 3774b | 33.22±0.62 | 35.76±0.39 | 46.72±0.51 | 52.07±1.33 | 38.26±0.00 | 42.39±0.90 | 58.70±1.31 | 61.57±1.05 | 687.71±8.95 | 709.28±4.43 | 119.91±2.82 | 120.95±3.47 |
| *Aegilops ovata* | 24a | 27.18±0.09 | 29.71±0.74 | 47.22±0.17 | 50.79±0.31 | 32.70±0.46 | 37.08±0.55 | 53.92±1.23 | 57.23±0.82 | 598.89±9.85 | 576.42±1.88 | 99.43±3.53 | 103.43±1.30 |
|  | 21b | 29.87±0.37 | 31.23±0.46 | 42.31±0.19 | 48.94±0.61 | 37.11±0.83 | 41.59±0.26 | 54.58±1.39 | 56.57±0.01 | 595.53±1.54 | 630.11±12.46 | 106.69±1.01 | 114.25±2.55 |
|  | 23 | 28.59±0.02 | 32.31±0.10 | 51.01±0.64 | 53.22±0.97 | 34.67±0.43 | 39.19±0.74 | 48.70±1.11 | 50.15±0.83 | 442.43±3.91 | 486.32±10.37 | 107.12±3.26 | 116.08±1.47 |
| *Aegilops peregrina* | PI 604192a | 28.19±0.43 | 29.71±0.57 | 35.62±0.10 | 38.16±0.01 | 30.34±0.13 | 36.86±0.71 | 42.04±0.60 | 46.27±0.73 | 585.32±7.40 | 597.86±12.81 | 98.62±4.49 | 107.19±0.11 |
|  | PI 603247 | 25.93±0.19 | 26.75±0.32 | 33.29±0.07 | 35.81±0.17 | 28.04±0.05 | 31.81±0.10 | 34.62±0.36 | 36.94±0.30 | 435.36±2.03 | 492.36±7.68 | 89.99±1.39 | 102.15±1.91 |
|  | PI 604149 | 27.37±0.69 | 28.62±0.16 | 32.01±0.53 | 34.01±0.48 | 26.63±0.46 | 35.50±0.49 | 41.08±0.04 | 42.94±0.73 | 422.07±7.90 | 483.93±1.76 | 92.53±3.42 | 98.08±1.07 |
|  | PI 604150 | 20.91±0.34 | 22.77±0.02 | 33.83±0.67 | 36.92±0.46 | 16.81±0.25 | 19.28±0.05 | 21.09±0.30 | 25.90±0.02 | 408.53±5.10 | 463.57±0.96 | 95.13±4.38 | 105.06±1.80 |
|  | PI 604176b | 15.71±0.39 | 17.58±0.10 | 44.15±0.99 | 45.26±0.92 | 39.50±0.72 | 40.12±0.79 | 46.66±0.36 | 51.37±0.21 | 624.82±6.18 | 688.39±11.46 | 91.13±4.07 | 94.52±1.27 |
|  | 55a | 16.46±0.16 | 18.05±0.28 | 39.88±0.77 | 41.25±0.02 | 37.37±0.39 | 38.73±0.14 | 43.33±0.31 | 48.82±0.25 | 505.05±1.89 | 543.21±8.19 | 102.26±1.17 | 109.17±2.67 |
|  | PI 604145b | 17.15±0.36 | 19.96±0.34 | 40.28±0.31 | 48.42±0.55 | 15.37±0.38 | 18.61±0.46 | 23.15±0.44 | 30.94±0.72 | 385.78±0.60 | 417.78±2.82 | 99.06±7.01 | 111.31±1.74 |
|  | 55b | 26.86±0.21 | 28.72±0.21 | 38.72±0.93 | 42.14±0.53 | 26.88±0.57 | 33.54±0.29 | 39.15±0.34 | 44.12±0.06 | 254.28±5.95 | 282.89±1.32 | 95.49±3.42 | 114.11±1.90 |
|  | PI 604168 | 18.21±0.23 | 19.22±0.32 | 34.52±0.04 | 36.61±0.32 | 24.07±0.19 | 28.51±0.05 | 29.58±0.46 | 32.51±0.47 | 435.39±11.10 | 466.64±3.40 | 92.33±3.72 | 100.99±1.78 |
|  | PI 603931 | 24.29±0.14 | 25.89±0.02 | 44.97±0.91 | 47.86±0.95 | 18.98±0.14 | 20.98±0.45 | 29.35±0.50 | 34.20±0.12 | 596.21±2.48 | 619.78±12.25 | 96.97±2.67 | 105.22±0.71 |
|  | 55c | 20.63±0.23 | 22.77±0.51 | 39.94±0.27 | 42.56±0.24 | 19.64±0.43 | 21.12±0.05 | 35.17±0.16 | 40.78±0.85 | 323.82±1.18 | 399.53±4.16 | 93.59±4.83 | 97.07±1.15 |
| Amphidiploid | ID 81/17 | 18.40±0.43 | 20.28±0.24 | 28.80±0.10 | 32.10±0.59 | 21.12±0.46 | 23.85±0.54 | 31.31±0.67 | 35.05±0.05 | 408.17±6.35 | 441.24±5.92 | 78.88±5.88 | 86.72±2.01 |
|  | EC 787010 | 16.92±0.13 | 19.04±0.24 | 33.13±0.45 | 38.40±0.86 | 20.57±0.16 | 25.04±0.16 | 23.66±0.55 | 26.02±0.17 | 387.50±8.87 | 334.28±1.39 | 83.85±1.03 | 88.56±1.44 |
|  | EC 787008 | 20.04±0.48 | 21.54±0.08 | 31.91±0.51 | 35.12±0.58 | 22.09±0.54 | 24.36±0.62 | 24.45±0.59 | 29.00±0.09 | 352.82±0.55 | 471.10±11.28 | 78.57±0.75 | 85.66±1.16 |
| *Triticum dicoccoides* | 102b | 18.09±0.24 | 21.71±0.16 | 35.93±0.36 | 37.49±0.13 | 21.55±0.39 | 25.35±0.24 | 35.08±0.29 | 37.57±0.39 | 468.23±7.23 | 491.43±1.45 | 86.36±0.49 | 92.04±2.41 |
|  | 13993a | 21.29±0.27 | 22.47±0.15 | 39.04±0.93 | 41.12±0.89 | 23.06±0.58 | 26.71±0.33 | 39.10±0.04 | 41.25±0.15 | 342.14±7.48 | 385.03±2.61 | 95.11±2.53 | 101.19±0.55 |
|  | 103a | 20.59±0.46 | 22.67±0.43 | 33.79±0.65 | 37.10±0.50 | 24.59±0.45 | 28.68±0.66 | 32.96±0.51 | 35.55±0.13 | 525.72±9.03 | 540.00±3.93 | 99.40±1.32 | 103.56±2.43 |

Table S2. Principal component analysis for antioxidant enzymes in different wheat species

| Enzymes | PC1 | PC2 | PC3 | PC4 | PC5 | PC6 |
| --- | --- | --- | --- | --- | --- | --- |
| CAT | -0.456 | 0.141 | 0.063 | -0.054 | 0.856 | 0.179 |
| APX | -0.427 | 0.360 | 0.273 | 0.550 | -0.161 | -0.534 |
| POX | -0.446 | -0.138 | 0.164 | -0.759 | -0.195 | -0.376 |
| GR | -0.459 | 0.232 | 0.117 | 0.008 | -0.443 | 0.725 |
| PAL | -0.277 | -0.880 | 0.185 | 0.331 | -0.009 | 0.067 |
| PPO | -0.352 | -0.055 | -0.920 | 0.092 | -0.082 | -0.108 |
| Eigenvalue | 4.046 | 0.829 | 0.581 | 0.248 | 0.186 | 0.109 |
| Variability (%) | 67.43 | 13.82 | 9.69 | 4.14 | 3.11 | 1.81 |
| Cumulative % | 67.43 | 81.25 | 90.94 | 95.08 | 98.19 | 100 |
